# The prevalence of functional gastrointestinal disorders related symptoms and the association with working place among healthcare workers who were in the fighting against COVID-19 in regional China

**DOI:** 10.3389/fpubh.2022.1048935

**Published:** 2022-12-15

**Authors:** Yan Zhang, Yue Guan, Ya Shen, Huifen Qiao, Jie Yuan, Fei Xu

**Affiliations:** ^1^Department of Gastroenterology, Nanjing First Hospital, Nanjing Medical University, Nanjing, China; ^2^Department of Integrated Service and Management, Jiangsu Provincial Center for Disease Control and Prevention, Nanjing, China; ^3^Department of Medical Psychology, Nanjing Brain Hospital Affiliated to Nanjing Medical University, Nanjing, China; ^4^Nanjing Municipal Center for Disease Control and Prevention, Nanjing, China; ^5^Department of Epidemiology, School of Public Health, Nanjing Medical University, Nanjing, China

**Keywords:** COVID-19, function gastrointestinal disorders related symptom, healthcare worker, prevalence, China

## Abstract

**Objectives:**

To investigate the prevalence of functional gastrointestinal disorders (FGIDs) related symptoms among healthcare workers (HWs) who were in the fighting against COVID-19 in Nanjing of China, and further to examine the association between working place and FGIDs-related symptoms among HWs during the period of COVID-19 epidemic.

**Methods:**

An online anonymous survey was conducted among those HWs without history of FGIDs, who took part in the fighting against the COVID-19 epidemic between July and September of 2021 in Nanjing, China. All the 15 FGIDs-related symptoms included in the Rome IV diagnostic questionnaire for adults were investigated in this study. The outcome variable was the presence of FGIDs-related symptoms (“Yes” or “No”), while the independent measure was participants' working place (“in-ward” or “out-ward”). Logistics regression models were applied to calculate odds ratios (ORs) and 95% confidence intervals (CIs) to assess the association of working place with FGIDs-related symptoms among those healthcare workers.

**Results:**

Totally, 336 eligible participants completed the survey. The prevalence of FGIDs-related symptoms was 48.8% (95%CI = 43.4%, 54.3%) among overall participants, with 40.7% (95%CI = 33.14%, 48.71%) and 56.3% (95%CI = 48.59%, 63.73%) for in-ward and out-ward HWs, respectively. Compared to their in-ward counterparts, those out-ward HWs were at a 1.88-fold likelihood (95%CI = 1.22, 2.89) to experience FGIDs-related symptoms during the period of fighting against the COVID-19 epidemic. After adjustment for potential confounders, such a positive association attenuated but still remained significant.

**Conclusions:**

A high prevalence of FGIDs-related symptoms was observed among those HWs who were without history of FGIDs during the fighting against COVID-19, and out-ward HWs were at a significantly higher risk to experience FGIDs-related symptoms relative to their in-ward counterparts in regional China. It has important implications that particular attention shall be paid to functional gastrointestinal issues for healthcare workers, especially those who are at uncertain risks of infectious diseases, when they participate in response to public health emergencies in future.

## Introduction

Functional gastrointestinal disorders (FGIDs) are non-organic diseases, usually referring to common syndromes with either upper or lower gastrointestinal (GI) symptoms, which are diagnosed based on the Rome IV criteria in clinical practice (1, 2). It has been well documented that psychosocial factors, including anxiety, stress, fatigue and depression, are the main risk factors of FGIDs (3–6). During the epidemic of COVID-19, elevated prevalence of FGIDs-related symptoms was observed among community residents than that under the usual circumstance before the outbreak (7). Moreover, an increased prevalence of FGIDs-related symptoms was also recorded within healthcare workers (HWs) who took part in the fighting against COVID-19 in China (8). The health states of healthcare workers are crucial for them to maintain good performance in health service activities/behaviors, so it is of particular interest to further investigate the occurrence of FGIDs-related symptoms among healthcare workers who are participating in the fighting against COVID-19.

Since the first wave of COVID-19 outbreak was contained in March of 2020, China has developed an effective management strategy, namely “zero tolerance for local transmission (dynamic zero-COVID)”, to tackle local outbreaks of COVID-19 (9). This strategy worked very effective for limiting the spread of COVID-19 within a small community or a city and then eliminating it completely in two or three latent periods, and consequently community residents lived relatively normal life in China (10). Based on this COVID-19 control strategy, healthcare workers involved in the fighting against COVID-19 were required to be managed with a closed-loop mode in China, which referred to a special system that, during the entire duration of fighting against COVID-19, all the healthcare workers: (1) would be accommodated in separate rooms of isolated hotels (one person, one room), (2) worked within a designated place (e.g., hospital wards, fever clinics, quarantine rooms, labs for testing SARS-CoV-2, or field sites for sample collection) and were not allowed to visit un-designated sites, (3) could not contact each other face-to-face without protective suits, and (4) would be quarantined for at least 2 weeks before returning to regular life (11).

For HWs, they were also vulnerable to SARS-CoV-2, even though they used personal protective equipment when they were fighting against COVID-19 (12, 13). Moreover, the HWs faced some psychological problems, including stress, depression, etc, brought by the SARS-CoV-2 epidemic (14). For Chinese HWs under the closed-loop management for fighting against COVID-19, they would face much more psychological issues than usual, e.g., fear and fatigue in addition to stress and anxiety (15, 16). Meanwhile, a positive association of these psychological factors with FGIDs-related symptoms was observed among healthcare workers during the period of fighting against COVID-19 in China (8). However, in the study on healthcare workers' psychological stress and FGIDs-related symptoms in China, participants were limited to physicians and nurses (in-ward HWs) who were responsible for treating COVID-19 patients in isolated hospital wards regardless of their FGIDs history (8). On the other hand, in addition to physicians and nurses who worked within hospital wards, there were a lot of healthcare workers involved in the fighting against COVID-19, including those who worked at fever clinics, quarantine rooms, labs for testing SARS-CoV-2 and field sites for specimen collection. Obviously, these healthcare workers (out-ward HWs) who took part in the fighting against COVID-19 at designated places outside hospital wards were also at a risk of SARS-CoV-2 and faced psychological stress too.

These two categories of healthcare workers would contact different subjects: in-ward HWs would directly contact the diagnosed COVID-19 patients in hospital wards, while out-ward HWs would closely reach those people who might be or might not be infected with SARS-CoV-2. These two types of HWs were at risks of COVID-19 with different natures. From psychological perspectives, in-ward HWs faced certain risks of COVID-19, while out-ward HWs had to face uncertain/unpredictable COVID-19 circumstances. Meanwhile, uncertainty might produce more stress, anxiety and fear for people compared to certainty (17). It has been observed among Israelis that those participants who could not ensure whether being infected with SARS-CoV-2 tended to report negative psychological emotions relative to those who were certain that they had been infected with the virus (18). Thus, it is reasonable to assume that the occurrence rate of FGIDs-related symptoms might be different between the in-ward and out-ward healthcare workers, as they, respectively, faced certain and uncertain risks of COVID-19 and thus suffered from different levels of psychological stress.

Although the “dynamic zero-COVID” strategy worked well, China still saw local outbreaks of COVID-19 occasionally. For example, a COVID-19 epidemic (Nanjing epidemic) occurred with 9 patients confirmed on July 20 of 2021 and lasted 19 days in Nanjing, China (19). The initial 9 patients were international air-flight cabin cleaners and finally 329 patients in total, infected with delta variant strains, were identified in the Nanjing COVID-19 epidemic (19). To fight against this epidemic, all the dispatched healthcare workers were administered with different tasks under the closed-loop management system from July 20 to September 2 in 2021 after a specific emergency response was activated (19).

To better understand the prevalence of FGIDs-related symptoms among healthcare workers who took part in the fighting against COVID-19, a study was conducted among those healthcare workers during the period of COVID-19 epidemic in Nanjing, China. The aims of this study were: (1) to investigate the prevalence of FGIDs-related symptoms among the healthcare workers who were without FGIDs history; and (2) to test the hypothesis that out-ward HWs were more likely to experience FGIDs-related symptoms compared to those in-ward HWs during the period of fighting against COVID-19 in regional China.

## Methods

### Study design and participants

This self-administered online anonymous survey was conducted on September 14 of 2021. The eligible participants were all the healthcare workers: (1) who took part in the fighting against Nanjing COVID-19 epidemic in 2021 and were willing to participate in the study, (2) who were under the closed-loop management, and (3) who reported no history of FGIDs. These healthcare workers included physicians, nurses, lab technicians and administrative staffs. All of them worked at hospital wards\ICUs, fever clinics, quarantine rooms, labs for testing SARS-CoV-2 or field sites for specimen collection according to their specific working tasks.

The sample size was estimated based on the study design, expected statistical power and estimated prevalence of FGIDs-related symptoms among healthcare workers without FGIDs history. The study was designed as a cross-sectional survey and statistical power was expected as 90%. Regarding the estimated prevalence of FGIDs-related symptoms, there was no figure available for healthcare workers without FGIDs history. Then, the prevalence of FGIDs-related symptoms observed among overall healthcare workers during COVID-19 pandemic in 2020 in Wuhan city (83.2%) and that among general Chinese adults (34.4%) could be used to presume the prevalence of FGIDs-related symptoms as ~48.8% (83.2–34.4%) among healthcare workers without FGIDs history (8, 20). Thus, with additional consideration of safety efficiency, the sample size was finally determined as ~290 in our study.

Written informed consent form was prepared as the second page of the online questionnaire. Each eligible participant would read this form and then decided whether or not to take part in the survey. If he/she was willing to join the survey, the participant must ensure he/she had read and understood all the information presented in the consent form. Otherwise, the survey could not be activated. This study was reviewed and approved by the Ethics Committee of The Affiliated Nanjing Hospital of Nanjing Medical University. All methods were performed in accordance with relevant guidelines and regulations based on the Declaration of Helsinki.

### Data collection

All the healthcare workers were classified into different working teams based on their working sites (one site, one team) during the period of fighting against Nanjing epidemic of COVID-19. The members within a working team were organized into a WeChat group for easy communication and coordination with each other (one working team, one WeChat group), resulting in totally 15 WeChat groups. Each team member was usually registered with only one WeChat group. However, some administrative staff and chief/senior professionals would be involved in two or more WeChat groups, as they were responsible for supervising different working teams with similar tasks. For example, one chief physician might be in charge of two or more COVID-19 patient wards.

The survey instrument was developed as an internet-based questionnaire using the platform of SO-JUMP, a free-to-use internet-based survey questionnaire provider in China (21). The online questionnaire collected information on participants' socio-demographic, years of professional employment, daily time with protective suit, working duration of involvement in the fighting against Nanjing COVID-19 epidemic, night shift, history of selected chronic diseases (diabetes, hypertension and COPD) and all the 15 FGIDs-related symptoms included in the Rome IV questionnaire. On the first page of the questionnaire, survey purposes, summary description of survey contents, inclusion criteria, the time it would take to complete, and attention points were described, while written informed consent was shown on page two. Only those participants who signed the consent form (ticked the option of “YES” to question: I have read and understood all the information presented in this consent form and am willing to participate in this study) could start and complete the entire survey. On the last page, each participant was still asked to make sure that he/she had properly responded to each question item, although each question was designed as compulsory item to warrant no missing answers. Only after completing this step, he/she was able to click the button “SUBMIT” to finish the survey.

**Figure 1:**
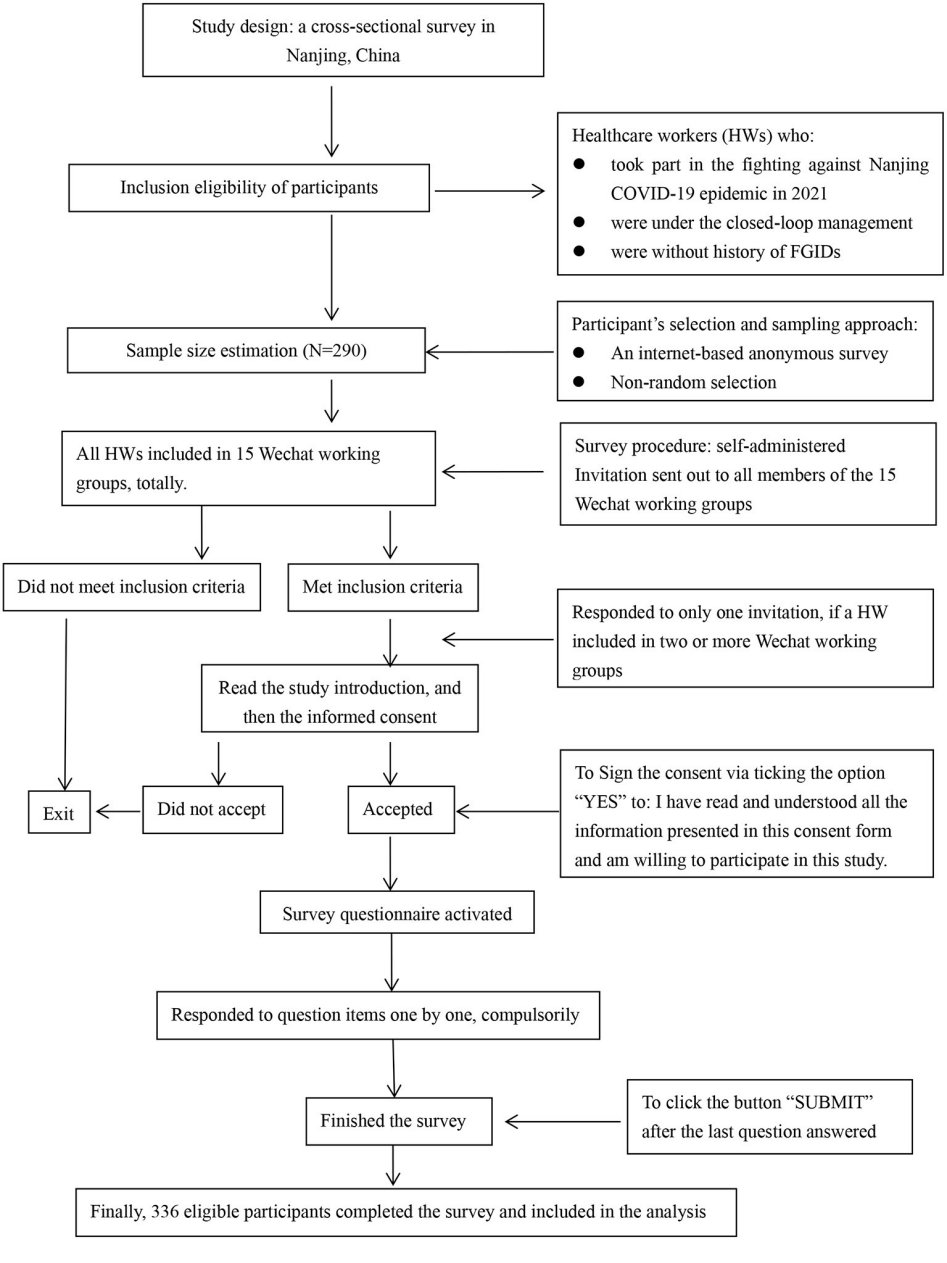
Flowchart of participant's selection and survey procedure in the study.

Additionally, considering that: (1) one healthcare worker might join in two or more WeChat groups, and (2) this was an anonymous survey, one reminding sentence was prepared as a separate short paragraph on page one. It reads that “Please kindly note that one person is expected to participate in this survey one time only. If you joined in two or more WeChat groups and received this survey invitation in different WeChat groups, please respond to it only once”. In this way, “one person, one response” would be maximally warranted in the present study. The survey was conducted on September 14, the second day that the last team left the closed-loop management for 2 weeks quarantine before returning to their regular job/life. On the survey day, the e-questionnaire was distributed to each WeChat group and all eligible members were invited to take part in the survey. The participants' selection and survey procedure were illustrated in Figure 1.

### Study variables

The Rome IV diagnostic questionnaire for adult FGIDs was developed for physicians to clinically diagnose FGIDs cases or investigators to define FGIDs cases in epidemiological surveys (22). For diagnosing/defining FGIDs cases, this Rome IV questionnaire includes totally 89 question items that consist of FGIDs-related symptoms, presence frequency of the FGIDs-related symptoms, onset time of FGIDs-related symptom, etc. (22). However, of these 89 questions in the Rome IV questionnaire, only 15 items ask about FGIDs-related symptoms regarding esophagus, stomach/intestines, gallbladder and pancreas, rectum and anal canal (22). In detail, these 15 FGIDs-related symptoms are: sensation of a lump or foreign body in the throat, dysphagia, retrosternal chest pain, heart burn, postprandial fullness, early satiation, early satiation that prevents finishing a regular meal, nausea, vomiting, reflux, belching, abdominal pain, constipation, diarrhea, bloating and incontinence (22). In our study, the purpose was to investigate the prevalence of FGIDs-related symptoms and to examine the association of working place with FGIDs-related symptoms among healthcare workers, not to determine the cases of FGIDs. Therefore, it was not necessary to include all the 89 question items of The Rome IV questionnaire in our study. It was appropriate that these FGIDs-related symptoms were used as outcome events in our survey. Each participant was asked to respond to the question “From the beginning you took part in the fighting against this wave of COVID-19 epidemic till you left the closed-loop management, during this whole time period, did you ever experience, at least once, any of the following symptoms?”. All participants were asked to carefully respond to the 15 symptom items one by one.

The outcome variable was the prevalence of FGIDs-related symptoms. The positive outcome event was defined as self-reported experience of any of the 15 FGIDs-related symptoms by participants when they took part in the fighting against Nanjing COVID-19 epidemic in 2021. Therefore, participants were classified as: “Experienced FGIDs-related symptoms (“Yes”)” or “Did not experience any FGIDs-related symptoms (“No”)” in the analysis.

Independent measure was working places that healthcare workers were assigned to during their involvement in the fighting against this wave of COVID-19 epidemic. Participants were then categorized into: “in-ward HWs” or “out-ward HWs” in our analysis. “in-ward HWs” referred to healthcare workers who were responsible for treating confirmed COVID-19 patients in isolated hospital wards/ICUs, while “out-ward HWs” were all those who worked at places other than hospital wards/ICUs, including fever clinics, quarantine rooms, labs for testing SARS-CoV-2 and field sites for specimen collection.

The covariates adjusted for in the analysis were participants' age (<30, 30–39 or 40+ years), gender (men or women), educational level (junior college, undergraduate or graduate), professional title (junior, medium-grade or senior), occupation (physician, nurse or others), years of professional employment (1–5, 6–10, 10–20, 20–30, or 31+ years), daily time with protective suit (2.0–2.9, 3.0–3.9, 4.0–4.9, or 5.0+ h), continuous working days of involvement in fighting against this epidemic (7, 14, 21, 30, or 31+ days), night shift (“Yes” or “No”), history of selected NCDs (“Yes” or “No”).

### Statistical analysis

The Chi-square test was applied to compare differences in working places and prevalence of FGIDs-related symptoms between participants by selected socio-demographic characteristics. The prevalence of FGIDs-related symptoms was reported as percentage and 95% confidence interval (95% CI). Three logistic regression models were introduced to compute odds ratios (OR) and 95% CIs for assessing the associations between working place and FGIDs-related symptoms. Model 1 was an unvariate analysis with working place as the independent variable only. Model 2 was a multivariate analysis with working place as the independent variable and adjustment for participants' age, gender and educational level. And model 3 was also a multivariate analysis with working place as the independent variable and further consideration of participants' occupation, professional title, years of professional employment, daily time with protective suit, continuous working days of involvement in fighting against Nanjing COVID-19 epidemic, night shift, history of selected NCDs in addition to those controlled for in Model 2. Two-sided statistical significance was set as *P* < 0.05. Data were entered using EpiData 3.1 (The EpiData Association 2008, Odense, Denmark), and analyzed with SPSS 21.0 (IBM Corp, Armonk, NY, USA).

## Results

In the study, totally 336 healthcare workers from all the 15 WeChat groups completed the survey. Due to the nature of anonymous survey, it was not possible for us to gather personal identifications from all WeChat members, and thus we had no information on those who did not participate in our survey. Consequently, we were not able to make comparison of the potential difference in personal characteristics between the respondents and those who did not take part in the survey. Table 1 presented the selected characteristics of participants in the study. Among the 336 respondents, there were 52.4% participants aged <30 years, 79.8% of women, 71.4% with undergraduate education, 62.8% with junior professional title and 67.0% of nurses. There was no difference in out-ward and in-ward participants in terms of age, gender, educational level and professional title, while the proportion of nurses was significantly higher among in-ward than out-ward HWs (*p* < 0.01).

**Table 1:** Selected characteristics of participants in the study.

		**Participants**		**Work places**				*p*-value^*^
				**Out-ward**		**In-ward**		
		***N***	**%**	***n***	**%**	***n***	**%**	
Overall		336	100	174	51.8	162	48.2	
Age (years)								
	<30	176	52.4	92	52.9	84	51.9	
	30–39	133	39.6	65	37.4	68	42.0	0.40
	40+	27	8.0	17	9.8	10	6.2	
Gender								
	Man	68	20.2	34	19.5	34	21.0	0.74
	Woman	268	79.8	140	80.5	128	79.0	
Educational level								
	Junior college	36	10.7	18	10.3	18	11.1	
	Undergraduate	240	71.4	120	69.0	120	74.1	0.37
	Graduate	60	17.9	36	20.7	24	14.8	
Professional title								
	Junior	211	62.8	105	60.3	106	65.4	
	Medium-grade	99	29.5	54	31.0	45	27.8	0.60
	Senior	26	7.7	15	8.6	11	6.8	
Occupation								
	Physician	78	23.2	55	31.6	23	14.2	
	Nurse	225	67.0	87	50.0	138	85.2	<0.01
	Others	33	9.8	32	18.4	1	0.6	

* Chi-square test.

Table 2 showed the prevalence of FGIDs-related symptoms among participants in the study. The overall prevalence of FGIDs-related symptoms was 48.8% (95%CI = 43.4%, 54.3%) among the study population. There was no difference in prevalence of FGIDs-related symptoms examined between participants' categories by age, gender and professional title. Participants who obtained graduate educational level tended to report FGIDs-related symptoms (junior college vs. undergraduate vs. graduate: 10.7 vs. 71.4 vs. 17.9%; *p* = 0.02), and the lowest prevalence of FGIDs-related symptoms was recorded among nurses (physician vs. nurse vs. others: 56.4 vs. 44.0 vs. 63.6%; *p* = 0.03). However, the difference in prevalence of FGIDs-related symptoms between participants' sub-groups by education and occupation became non-significant after all the covariates were adjusted for in our analysis.

**Table 2:** The prevalence of FGIDs-related symptoms among participants in the study.

		**Participants**		**FGIDs-related symptoms**				*p* value^*^
				**Experienced**		**Did not experience**		
		***N***	**%**	***n***	**%**	***n***	**%**	
Overall		336	100	164	48.8	172	51.2	
Age (years)								
	<30	176	52.4	79	44.9	97	55.1	
	30–39	133	39.6	72	54.1	61	45.9	0.27
	40+	27	8.0	13	48.1	14	51.9	
Gender								
	Man	68	20.2	27	39.7	41	60.3	0.09
	Woman	268	79.8	137	51.1	131	48.9	
Educational level								
	Junior college	36	10.7	11	30.6	25	69.4	
	Undergraduate	240	71.4	117	48.8	123	51.2	0.02
	Graduate	60	17.9	36	60.0	24	40.0	
Professional title								
	Junior	211	62.8	96	45.5	115	54.5	
	Medium-grade	99	29.5	54	54.5	45	45.5	0.29
	Senior	26	7.7	14	53.8	12	46.2	
Occupation								
	Physician	78	23.2	44	56.4	34	43.6	
	Nurse	225	67.0	99	44.0	126	56.0	0.03
	Others	33	9.8	21	63.6	12	36.4	

* Chi-square test.

Table 3 displayed the association between working place (out-ward vs. in-ward) and FGIDs-related symptoms among study participants. Among the 162 in-ward HWs who were at certain risks of COVID-19, 40.7% (95%CI: 33.14%, 48.71%) reported the presence of FGIDs-related symptoms, while 56.3% (95%CI: 48.59%, 63.73%) ever experienced FGIDs-related symptoms within those 174 out-ward HWs who faced uncertain risks of COVID-19. Those out-ward HWs were at a 1.88-fold likelihood (95%CI: 1.22, 2.89) to experience FGIDs-related symptoms compared to their in-ward counterparts. Furthermore, after adjustment for potential confounders, such a positive relationship between working place (out-ward vs. in-ward) and FGIDs-related symptoms attenuated but still remained significant (Model 2: OR = 1.85, 95%CI = 1.19, 2.89; Model 3: OR = 1.77, 95%CI = 1.01, 3.13) among participants in the study.

**Table 3:** The association between work places (out-ward/in-ward) and presence of FGIDs-related symptoms among participants in the study.

		**Proportion of participants who experienced FGIDs-related symptoms (% and n/N)**	**Presence of FGIDs-related symptoms**					
			**Model 1^a^**		**Model 2^b^**		**Model 3^c^**	
			**OR**	**95% CI**	**OR**	**95% CI**	**OR**	**95% CI**
Work places where participants took	In-ward	40.7 (66/162)	1		1		1	
part in the fighting against COVID-19^*^	Out-ward	56.3 (98/174)	1.88	1.22, 2.89	1.85	1.19, 2.89	1.77	1.01, 3.13

a Model 1: uni-variate logistic regression analysis with work mode as the independent variable.

b Model 2: multi-variate logistic regression analysis, with adjustment for participants' age, gender and educational level.

c Model: multi-variate logistic regression analysis, with consideration of professional title, occupation, years of professional employment, daily time with protective suit, continuous working days of involvement in fighting against Nanjing COVID-19 epidemic, night shift, history of NCDs in addition to those adjusted for in Model 2.

* In-ward participants directly contacted with COVID-19 patients, while out-ward participants closely contacted those might be or might not be COVID-19 patients.

## Discussion

In this internet-based population study, we aimed to investigate the prevalence of FGIDs-related symptoms and to examine the association between working place and FGIDs-related symptoms among healthcare workers who were involved in the fighting against COVID-19 in the summer of 2021 in Nanjing of China. It was observed that, overall, 48.8% of the healthcare workers ever experienced at least one FGIDs-related symptom. Moreover, it was also examined that those who worked outside hospital wards were more likely to experience FGIDs-related symptoms relative to their counterparts who were within hospital wards responsible for treating COVID-19 patients.

It is really difficult for us to make comparison of the prevalence of FGIDs-related symptoms between our study and others, as there are very few similar studies available investigating FGIDs-related symptoms among HWs and particularly no previous studies, from the perspective of population-based occupational health, examining the potential influence of working place on FGIDs-related symptoms for HWs when they were in the fighting against COVID-19. Only one similar study was conducted in Wuhan city of China to investigate FGIDs-related symptoms among HWs who were involved in treating COVID-19 patients in early 2020 (8). And, one study was implemented in Bulgaria to examine changes in the prevalence of FGIDs-related symptoms due to COVID-19 pandemic among community residents (7). Although it is difficult to make direct comparison of findings between our study, the Wuhan and the Bulgaria studies, it is still important to make a broad comparison between them in order to help potential readers easily understand and prudently interpret the findings in our study.

Based on a latest report published in 2020, the overall FGIDs prevalence was documented as 40.3% (95%CI: 39.9%, 40.7%) among general adults worldwide and 34.4% (95%CI: 32.7%, 36.1%) in China (20). Moreover, during the COVID-19 epidemic period, a 12.9 percentage increase in the prevalence of FGIDs-related symptoms was observed from the summer (data collected during May and August) in 2019 (before the onset of COVID-19) to the early summer (data gathered during May and June) of 2020 (after the COVID-19 lockdown) among community adult population in Bulgaria (7). Furthermore, a cross-sectional study was conducted to investigate FGIDs-related symptoms among physicians and nurses who worked in hospital wards for treating COVID-19 patients in Wuhan of China in early 2020, showing that the overall prevalence of FGIDs-related symptoms was 83.2% among those healthcare workers (8).

The Bulgaria, Wuhan and our studies all reported the prevalence of FGIDs-related symptoms among participants based on symptoms included in the Rome IV diagnostic questionnaire. However, there were some differences between them. First of all, participants were different in these studies. In the Bulgaria study, participants were community adult population. In Wuhan survey, participants were limited to those physicians and nurses who worked in hospital wards (in-ward HWs only). However all the health workers who took part in the fighting against COVID-19 were recruited in our investigation, including not only physicians and nurses but also lab technicians and administrative staff. Moreover, different from that in Wuhan study, the participants in our survey worked not only in hospital wards but also at fever clinics, quarantine rooms, labs for testing SARS-CoV-2 or specimen collection sites. Second, those with FGIDs history were not excluded from the surveys in either Bulgaria or Wuhan study, while only those without history of FGIDs were eligible to take part in our study. Next, survey time also differed between these three studies. Both Bulgaria and Wuhan studies were implemented in early 2020, and at that time COVID-19 epidemic was still at the early stage, while our survey was conducted in mid-September of 2021, one and half a year later.

With respect to Wuhan and our Nanjing study, the figures of FGIDs-related symptoms prevalence were also different, 83.2% in Wuhan study (8) and 48.8% in our survey among overall participants. However, both the healthcare workers with and without FGIDs history were included in Wuhan study, while only those without FGIDs history were involved in our survey. Considering that the prevalence of FGIDs was about 34.4% among general adult population in China (20), the prevalence of FGIDs-related symptoms among those in-ward healthcare workers might be estimated as around 48.8% (83.2−34.4%) if the study participants were limited to healthcare workers without FGIDs history in Wuhan study, which was just slightly higher than that (40.7% = 66/162) observed among in-ward healthcare workers in our study.

When Nanjing COVID-19 epidemic occurred in July of 2020, the closed-loop management system had been well-established in China and the transmission characteristics of SARS-CoV-2 and treatment of COVID-19 patients were also further understood (10). Moreover, based on psychological insights, a negative event, such as COVID-19 epidemic, may affect people's emotion more strongly at the onset stage than some time later, as the extinction of fear-related emotion will actively occur with time going on (23, 24). This was also supported by findings from two population-based longitudinal studies during COVID-19 epidemic period (25, 26). One of them was conducted among general population with the first survey in early 2020 in China, documenting that community residents tended to report slightly but significantly lower scores for post-traumatic stress disorder (PTSD) symptoms 4 weeks later after the baseline survey (25). The other survey was implemented among healthcare workers with the first survey in May and the second in November of 2020 in Spain, showing that HWs experienced a significant improvement in stress-related symptoms over the 6-month follow-up period (26). Thus, it was applauded that the emotional/mental status (e.g., fear, stress) of healthcare workers involved in the fighting against Nanjing COVID-19 epidemic in mid-September of 2021 was still affected by COVID-19 but with a weakened extent.

In our study, one of the main findings was that a high prevalence of FGIDs-related symptoms was observed among overall HWs without FGIDs history. As COVID-19 is an emerging acute respiratory infectious disease, people initially have no clear idea on its transmission characteristics and treatment approaches (27). It could negatively affect the emotional/mental conditions, yielding stress, fear and anxiety, during the period of its epidemic for not only community residents but also healthcare workers (15, 28, 29). Meanwhile, stress, fear and anxiety were the main influencing factors of FGIDs (4, 5). Thus, it could, at least in part, to explain that the high prevalence of FGIDs-related symptoms was observed among healthcare workers without FGIDs history during COVID-19 epidemic in our study.

Another interesting and important finding in our study was that out-ward HWs were more likely to experience FGIDs-related symptoms compared to their in-ward counterparts who were within hospital wards responsible for treating COVID-19 patients. Those out-ward HWs were at uncertain risks of COVID-19, as they had close contacts with many people who might be or might not be infected with SARS-CoV-2. On the other hand, the in-ward HWs were at certain risks of COVID-19, as they just directly contacted these confirmed COVID-19 patients within well-equipped isolated hospital wards/ICUs. From the psychological perspective, uncertainty makes it difficult for people to prepare properly for unpredictable future negative events, and consequently it might produce more anxiety, stress and fear for people relative to certainty (17, 18). This might partly explain that out-ward HWs at uncertain risks of COVID-19 tended to experience FGIDs-related symptoms compared to their in-ward counterparts who were at certain COVID-19 risks.

This is the first study investigating FGIDs-related symptoms among healthcare workers who were without history of FGIDs and involved in the fighting against a COVID-19 epidemic in regional China. There were some strengths of this study. Firstly, participants were recruited from all healthcare workers who were responsible for different specific tasks against a COVID-19 epidemic. Secondly, all of the participants were without history of FGIDs, which provided a deep insight into the potential impact of COVID-19 on the occurrence of FGIDs-related symptoms. Finally, interesting findings were examined in that a high prevalence of FGIDs-related symptoms was observed among healthcare workers who were in the fighting against COVID-19, and, moreover, those out-ward HWs tended to experience FGIDs-related symptoms relative to their in-ward counterparts.

However, limitations also should be mentioned. Firstly, as this was an anonymous survey, we could not estimate the exact number of eligible participants and thus could not calculate the response rate. Thus, potential bias regarding participant's selection existed in this study. Secondly, also due to the nature of anonymous survey, it was not possible for us to identify the healthcare workers who were registered with two or more WeChat groups and those with FGIDs history. We then could not make comparison in main characteristics between the respondents and those who did not respond to the survey. Thirdly, the survey questionnaire was internet-based and self-administered in the study. Some eligible participants might not join in the study as they were not familiar with such a survey way. And, next, FGIDs-related symptoms not FGIDs cases were investigated among healthcare workers in the study, as this investigation was not developed as a questionnaire-based clinically-diagnostic study. Therefore, the findings from this study should be interpreted prudently.

It is meaningful to sum up the findings in the present study. The prevalence of FGIDs-related symptoms was observed as 48.8% among these HWs who were without history of FGIDs during the period of fighting against COVID-19 epidemic in regional China. There was no difference in the prevalence of FGIDs-related symptoms among participants by age and gender, separately. Moreover, 40.7% in-ward and 56.3% out-ward HWs, respectively, reported ever experiencing FGIDs-related symptoms. Furthermore, those out-ward HWs were at a significantly higher risk of experiencing FGIDs-related symptoms compared to their in-ward counterparts (adj.OR = 1.77). This study added the following values to literature. For HWs who are in the fighting against COVID-19, they are at an elevated risk of experiencing FGIDs-related symptoms, even if they have no history of FGIDs; and working outside hospital wards for tackling COVID-19 may exert more impact on the likelihood for HWs to experience FGIDs-related symptoms relative to working within hospital wards from the occupational health perspective.

## Conclusions

A high prevalence of FGIDs-related symptoms was observed among healthcare workers without FGIDs history during the period when they were involved in the fighting against COVID-19, and out-ward healthcare workers were more likely to experience FGIDs-related symptoms compared to their in-ward counterparts in regional China. It has important implications that particular and close attention shall be paid to functional gastrointestinal issues for healthcare workers, especially those who are at uncertain risks of infectious diseases, when they take part in response to public health emergencies in future.

## Data availability statement

The original contributions presented in the study are included in the article/supplementary material, further inquiries can be directed to the corresponding author/s.

## Ethics statement

The studies involving human participants were reviewed and approved by the Ethics Committee of Nanjing First Hospital, Nanjing Medical University, China. The patients/participants provided their written informed consent to participate in this study.

## Author contributions

YZ, JY, and FX conceived, designed, and directed the study. YZ, YG, and JY performed the experiments. FX analyzed the data. YZ, YG, YS, HQ, JY, and FX wrote the manuscript text. All authors critically reviewed the manuscript and approved the submission.
